# Effects of Preoperative Sleep Disorders on Anesthesia Recovery and Postoperative Pain in Patients Undergoing Laparoscopic Gynecological Surgery under General Anesthesia

**DOI:** 10.1155/2022/7998104

**Published:** 2022-12-15

**Authors:** Sicong Yu, Yicheng Xiong, Guangtao Lu, Xiangqing Xiong

**Affiliations:** ^1^Department of Anesthesiology, The First Affiliated Hospital of Wenzhou Medical University, Wenzhou, China; ^2^Department of Anesthesiology, Taizhou Central Hospital (Taizhou University Hospital), Taizhou, China; ^3^Alberta Institute, Wenzhou Medical University, Wenzhou, China

## Abstract

Sleep disorder dramatically affects people's physical and mental health. Here, we investigated the effect of preoperative sleep disorders on anesthesia recovery and postoperative pain in patients undergoing laparoscopic gynecological surgery under general anesthesia. 120 patients who underwent elective laparoscopic gynecological surgery under general anesthesia in Taizhou Central Hospital from November 2021 to March 2022 were included. According to the score of the Pittsburgh sleep quality index (PSQI), the participating patients were divided into four groups: control group (control group), mild sleep disorder group A (group A), moderate sleep disorder group B (group B), and severe sleep disorder group C (group C), with 30 patients in each group. The changes of mean arterial pressure (MAP) and heart rate (HR) at different time points, operation time, anesthesia time, extubation time, the time when Aldrete score reached 10 points, visual analog score (VAS) serum interleukin-6 (IL-6), interleukin-10 (IL-10), and tumor necrosis factor-*α* (TNF-*α*) were compared among different groups. Our study demonstrated that there were no significant differences in MAP and HR among the four groups at the same time points (all *P* > 0.05). Significant differences in the time of extubation and Aldrete score reaching 10 points had been found among the four groups (all *P* < 0.001), indicating more sleep disorder induced longer extubation and recovery time. There were significant differences in VAS scores among the four groups at both different and the same time points (all *P* < 0.001), suggesting more sleep disorders induced more pain in the sufferers. Serum IL-6 levels were significantly higher in the three sleep disorder groups than the control group at 6 h and 24 h after the operation (all *P* < 0.05), while group C has the highest IL-6 levels as compared to the other group (*P* = 0.09 and *P* < 0.001, respectively). At 6 h after operation, serum levels of TNF-*α* in group C were significantly higher than in the control group (*P* = 0.044), but no significant differences were found in the other two groups (all *P* > 0.05). Positive correlation with preoperative PSQI score has been found with the times of extubation, the time of Aldrete score reaching 10 points, the VAS at 1 h, 6 h, and 24 h after operation, the level of serum IL-6 at 1 day before operation and 6 h and 24 h after operation, and the TNF-*α* at 6 h and 24 h after operation (all *P* < 0.001). The present study showed that the degree of preoperative sleep disorders could affect the quality of postoperative awakening and pain of patients undergoing laparoscopic gynecological surgery under general anesthesia, which might be associated with the aggravation of inflammatory reactions in the body.

## 1. Introduction

According to the World Health Organization, 27% of the world's population suffers from sleep problems. Data from the Chinese Sleep Research Society show that the number of people with sleep disorders in China is as high as 300 million. The incidence rate among adults is as high as 38.2%. Sleep disorders can be associated with the occurrence and development of various diseases, such as hypertension, obesity, and other metabolic diseases, as well as cardiovascular and cerebrovascular diseases [1, 2].

Patients with sleep disorders usually have a nocturnal awakening and daytime drowsiness, and some patients have a history of taking sleeping pills. Multiple studies have confirmed that long-term sleep disturbance may impair cognitive function, prolong postoperative recovery time, have a higher risk of complications, and even cause postoperative delirium in severe cases [3–5]. Among the influencing factors of postoperative pain, preoperative sleep disturbance is found to be crucial, which may reduce the pain threshold, enhance pain sensitivity, and affect the postoperative rehabilitation process [6, 7]. In addition, sleep disorders can lead to the body's inflammatory mechanisms regulating exceptions. The primary manifestation is the imbalance of inflammatory factor levels, such as interleukin 6 (IL-6), interleukin 10 (IL-10), tumor necrosis factor-alpha (TNF-*α*), and c-reactive protein (CRP). The degree of sleep disorders may be related to serum inflammatory factors [8, 9]. However, whether sleep disorders of different degrees will affect the quality of postoperative recovery and pain is still insufficient, there still have been numerous studies reporting that women are at high risk for poor sleep quality [10].

The positive relationship between sleep and surgery has been investigated in recent years. For example, it was reported that postoperative sleep disorders were more affected by evening operations than by morning operations. And some investigators found that sleep quality plays a critical role in influencing the recovery of surgical patients. Since perioperative medical care developed, sleep quality and sleep patterns have become increasingly important. At present, however, there is a paucity of literature on preoperative sleep pattern in surgical patients, especially in the female patients, and these data are limited. Therefore, we selected gynecological patients for discussion in this study to help clinicians to provide patients' individualized intervention during the perioperative period, to improve the quality of postoperative recovery, reduce postoperative pain, and promote rapid recovery.

## 2. Materials and Methods

### 2.1. Participants

The ethics committee approved this experiment at Taizhou Central Hospital (Affiliated Hospital of Taizhou University) (Ethical code: 2021 L-11-08). This research studied 120 patients who underwent elective laparoscopic gynecological surgery under general anesthesia in Taizhou Central Hospital from November 2021 to March 2022. Supplementary Figure 1 showed the CONSORT flow chart for patient enrollment. All patients and their responsible family members were fully aware of this study and signed informed consent.

This study included patients matching the following criteria:

- Age between18 and 75 years old
- Body mass index (BMI) between 18.5 and 30 kg/m2
- American Society of Anesthesiologists (ASA) grade I ~ II
- Operation time between 1 h and 3 h
- No hypertension, diabetes, and heart disease before the operation
- Blood routine examination, coagulation index, and relevant preoperative examinations are all regular
- Preoperative visual analog scale (VAS) of 0

Meanwhile, patients with the following criteria were excluded:

- Transferred to laparotomy or admitted to the Intensive Care Unit (ICU) unplanned after laparoscopic surgery
- Undergoing emergency surgery
- Hypertension and diabetes before surgery
- With a history of coronary heart disease, cerebral hemorrhage, brain trauma, epilepsy, or liver and/or kidney dysfunction
- Previous central nervous system diseases
- Mental illness history or taking drugs for a long time
- Alcohol and/or drug abuse history
- Parkinson's disease, Alzheimer's disease, and delirium
- Severe sensory organ defects (such as vision or hearing impairment) or unable to communicate normally
- The case data are incomplete

### 2.2. Anesthesia

Before induction, patients of the four groups were intravenously injected with 4 ug/ml dexmedetomidine at a loading dose of 1 ug/kg for 10 minutes. Then, they were induced by sufentanil 0.5 ug/kg, rocuronium 1 mg/kg, and propofol 2 mg/kg. Immediately, after successful intubation, patients were ventilated with an oxygen and air mixture (FiO2 = 0.4) and maintained pressure of end-tidal CO_2_ (PetCO_2_) at 30-45 mmHg. During the operation, anesthesia was maintained with 1-2% sevoflurane, remifentanil (0.5 ug/kg/h), and dexmedetomidine (0.3 ug/kg/h). Rocuronium was added according to the time. Patients were transferred to the postanesthesia care unit (PACU) when awake, and the endotracheal tubes were removed after the operation.

### 2.3. Grouping Method

All participating patients were evaluated for sleep quality by The Pittsburgh Sleep Quality Index (PSQI) [11] after admission. This scale was designed and proposed by Buysse et al. at the University of Pittsburgh in 1989. It assessed the sleep quality of the subjects over the past month. There are nine questions in total. Questions 1-4 are blank-filling questions, and questions 5-9 are multiple-choice questions, of which question 5 consists of 10 subsequent questions. This scale contains an overall score of 21, with higher scores indicating poorer sleep quality. The patients are expected to answer all the questions in the scale within 5-10 minutes.

According to PSQI score results, each group included thirty patients following the sample size 1 : 1 : 1 : 1 matching. The PSQI score of 0 ~ 5 indicated good sleep quality and was included in the control group. Those with scores of 6-10 indicated that the sleep quality was fine and was included in the sleep disorder group A (group A). Those with scores of 11-15 considered that the sleep quality was not good and was included in sleep disorder group B (group B). Scores of 16 to 21 indicated poor sleep quality and were included in sleep disorder group C (group C).

### 2.4. Observation Indicator

The differences in the following indicators before and after surgery were compared among the four groups:

- General information including age, BMI, ASA grade, and preoperative PSQI score
- Changes in vital signs and surgical conditions: mean arterial pressure (MAP) and heart rate (HR) on a preoperative day one, T1 (at the time of entry), T2 (at the end of the operation), T3 (at the time of extubation), operation time, and anesthesia time
- Quality of postoperative recovery: extubation time, when Aldrete score reaches 10, the incidence of postextubation complications (hypoxemia, nausea, vomiting, respiratory depression or reintubation, chilliness, and bradycardia), and length of hospital stay. Aldrete score is mainly used for observation and evaluation of anesthesia recovery, including activity, respiration, blood pressure, consciousness, and oxygen saturation. Patients can leave the anesthesia recovery room after reaching 10 points
- Postoperative pain: VAS was used to evaluate the degree of pain at 1 h, 6 h, and 24 h after the operation
- Changes in inflammatory factors: peripheral venous blood was collected to detect the levels of IL-6, IL-10, and TNF-*α* one day before the operation and 1 h, 6 h, and 24 h after the operation
- The correlation between the above indicators and the preoperative PSQI score was analyzed

### 2.5. Statistics

SPSS22.0 statistical software was used for data analysis. The measurement data consistent with normal distribution were described by mean ± standard deviation. An intragroup comparison was performed by one-way repeated measure ANOVA. Spearman correlation analysis was conducted for detecting the correlation between two continuous variables. One-way ANOVA was used for comparison between multiple groups, while a quartile was used for data that did not conform to normal distribution. Kruskal-Wallis rank-sum test was used for comparison between groups, and the Bonferroni method was used for further comparison between pairs. Counting data were described by frequency and percentage. The Chi-square test or Fisher's exact probability method was used for comparison between groups. *P* < 0.05 indicated statistically significant differences.

## 3. Results

### 3.1. The General Information

Cases were collected from November 2021 until 30 patients were collected in each group. In the recruiting phase, 167 participants were assessed for eligibility, while 120 of them were finally included (Supplementary Figure 1). Among the 120 patients, the ages ranged from 26 to 68 years, with an average of 49.53 ± 10.94 years. There were 71 cases of ASA grade I and 49 cases of ASA grade II. BMI was 18.5-30.0 kg/m^2^, with an average of 23.73 ± 2.66 kg/m^2^. There were no statistically significant differences in age, BMI, and ASA grade among the four groups (*P* > 0.05). There were statistically significant differences in preoperative PSQI scores among the four groups (*P* < 0.05) (Table 1).

### 3.2. Changes in Vital Signs and Surgical Conditions

As shown in Table 2, the one-way repeated measure ANOVA showed significant differences in MAP and HR between the four groups at different time points (*P* < 0.05), displaying a trend of increasing, then decreasing, and subsequently increasing. There was no statistically significant difference in MAP and HR between the four groups simultaneously (all *P* > 0.05). There were no statistically significant differences in operation time and anesthesia time among the four groups (all *P* > 0.05).

### 3.3. Quality of Postoperative Recovery

**Figure 1:**
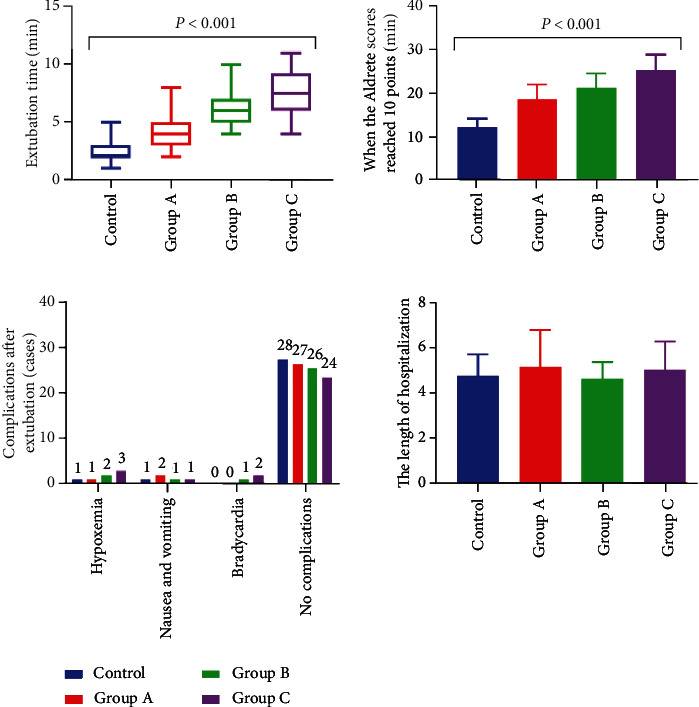
Comparison of the postoperative recovery quality among the four groups (*n* = 30).

Significant differences in the time of extubation and Aldrete score reaching 10 points had been found among the four groups (*P* < 0.05). Further, pairwise comparison showed that the two time periods in the control group were significantly shorter than those in the other three groups. The two time periods of group B were significantly longer than that in group A, while that of group C was significantly longer than that in group B (*P* < 0.05). There was no significant difference in complications after extubation and the length of hospitalization among the four groups (*P* > 0.05) (Figure 1).

### 3.4. Postoperative Pain

The one-way repeated measure ANOVA showed that VAS scores of the four groups at different time points were significantly different (*P* < 0.05), with a trend of first increasing and then decreasing. In line with this finding, VAS scores of the four groups at the same time were statistically significant (*P* < 0.05).

**Figure 2:**
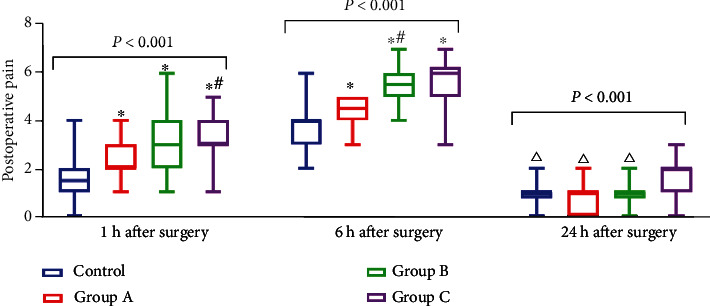
Comparison of postoperative pain (*n* = 30). Note: compared with the control group, ^∗^*P* < 0.05; compared with sleep disorder group A, #*P* < 0.05; compared with sleep disorder group C, △*P* < 0.05.

Further, pairwise comparison showed that at 1 h after the operation, patients in the control group had a lower VAS score than those in other groups (*P* < 0.05), and the patients in group A had a lower VAS score than those in group C (*P* < 0.05). Nevertheless, there was no significant difference between group A and group B and between group B and group C (*P* > 0.05). Similarly, the VAS score of the control group was significantly lower than that of other groups at 6 h after operation (*P* < 0.05), of which group A was significantly lower than that in groups B and C (*P* < 0.05). At the same time, there was no significant difference between group B and group C (*P* > 0.05). Higher VAS scores were observed 24 h after operation in group C as compared to the control group (*P* < 0.05), while there was no statistical significance among the other groups (*P* > 0.05) (Figure 2).

### 3.5. Inflammatory Factor Level

The one-way repeated measure ANOVA demonstrated significant differences in serum IL-6, IL-10, and TNF-*α* levels among the 4 groups at different time points (all *P* < 0.05). The levels of serum IL-6 and IL-10 were firstly increasing and then decreasing, while the levels of serum TNF-*α* were continuously decreasing.

There was no significant difference in serum IL-6 levels among the four groups at 1 d before and 1 h after the operation (*P* > 0.05) but statistically significant differences in serum IL-6 levels among the four groups at 6 h and 24 h after the operation (*P* < 0.05). The further pairwise comparison found that the serum IL-6 level of group B and group C was significantly higher than that of the control group at 6 h and 24 h after the operation (*P* < 0.05). The serum IL-6 level of group C was significantly higher than that of group A (*P* < 0.05), but there was no statistical significance between the other pairs of groups (*P* > 0.05).

Regarding serum IL-10, there was no significant differences among the four groups at 1 d before surgery, 1 h after operation, 6 h after operation, and 24 h after operation (*P* > 0.05).

**Figure 3:**
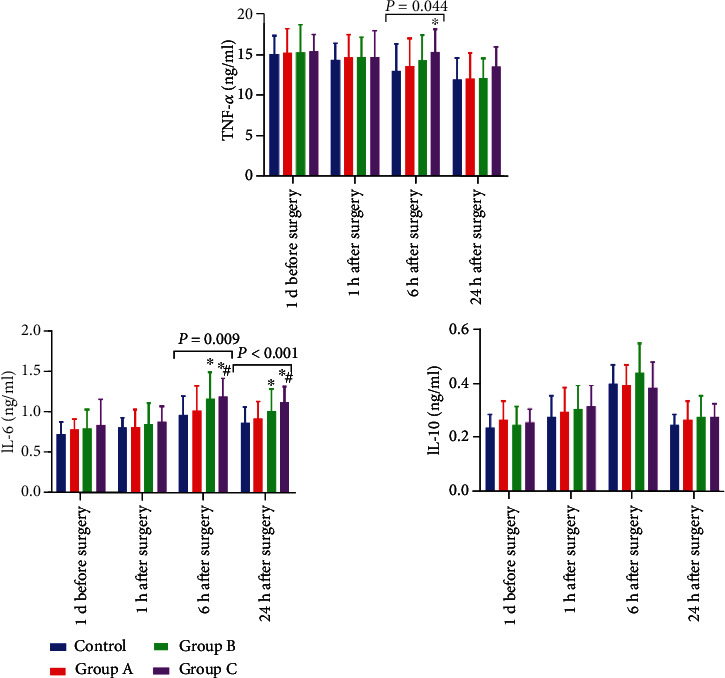
Comparison of inflammatory factor level (*n* = 30). Note: compared with control group, ^∗^*P* < 0.05; compared with group A, #*P* < 0.05.

Similarly, no significant difference in serum TNF-*α* was observed among the four groups on one day before the operation, 1 h after the operation, or 24 h after the operation. However, 6 h after the operation, the serum TNF-*α* levels in the four groups were statistically significant (*P* < 0.05). Further, pairwise comparison showed that the serum TNF-*α* levels of group C were significantly higher than that of the control group (*P* < 0.05), but there was no statistically significant difference among the other sleep disorder groups (*P* > 0.05) (Figure 3).

### 3.6. Correlation between Each Indicator and Preoperative PSQI Score

**Figure 4:**
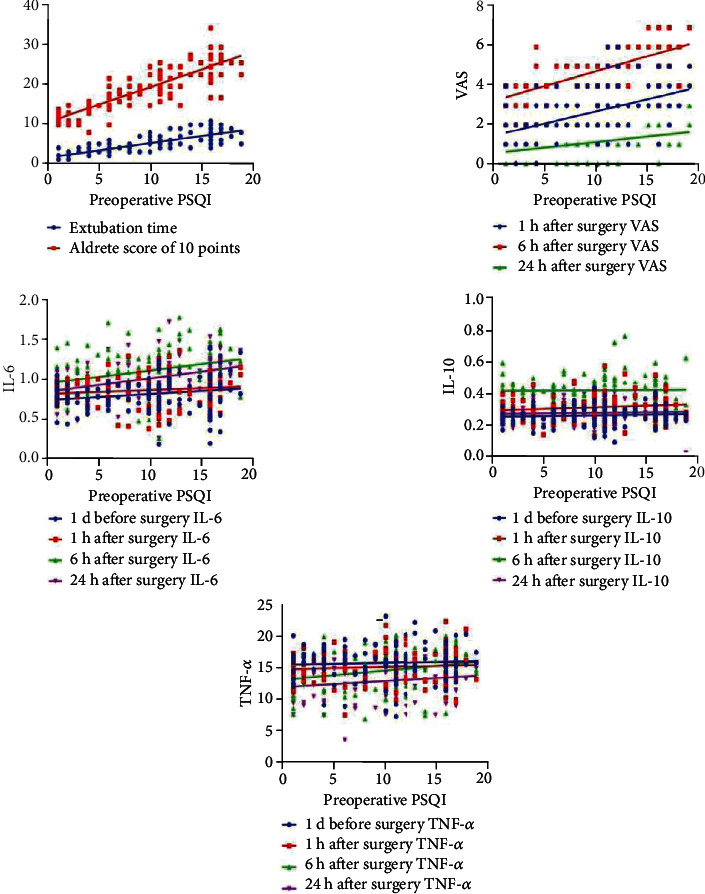
Correlation between each indicator and preoperative PSQI score.

As displayed in Figure 4, some scaling metrics were positively correlated with preoperative PSQI scores, such as extubation time, the time when Aldrete score reached 10 points, VAS scores at 1 h, 6 h, and 24 h after operation, serum IL-6 level at 1 d before the operation, 6 h and 24 h after operation, and TNF-*α* level at 6 h and 24 h after operation (all *P* < 0.05).

## 4. Discussion

Sleep is an essential physiological activity to maintain the normal physiological function of the human body. It is the resting state of the body to eliminate fatigue and the process of neural regulation of the central nervous system. Studies have proved that sleep directly regulates the immune system [12]. When sleep was disrupted, the body's endocrine disorder and the balance of the immune system should have changed, and the level of cytokines in the peripheral blood and the number of immune cells would have changed, resulting in a series of diseases [13]. However, the mechanism of sleep disorders has not been thoroughly studied. The possible related mechanisms include sleep-wake [14] and cytokine mechanisms. It can be found in many researches that a few sleep-related cytokines, e.g., IL-1, IL-6, IL-8, and TNF-*α*, have been extensively studied [15]. In this study, the concentrations of IL-6, TNF-*α*, and IL-10 were monitored before and after operation in patients with sleep disorders at different time points, to observe the relationship between sleep disorders and the level of cytokines. IL-6 and TNF-*α* are proinflammatory factors which promote the occurrence of the inflammatory response, while IL-10 is an inflammatory and immunosuppressive factor. Several studies [8, 16] reported that IL-6 and TNF-*α* significantly increase in patients with sleep disorders. Some other studies have shown [17] that IL-6 level is significantly elevated in insomniacs during the afternoon and evening hours. In addition, the concentrations of IL-6 and TNF-*α* have been proven to be positively correlated with Pittsburgh Sleep Quality Index (PSQI) scores [18], which is consistent with the results of this study.

However, researches on IL-10 are still controversial to some extent presently. In this study, no difference in IL-10 had been found, which may be due to the imbalance between proinflammatory factors and anti-inflammatory factors in the body.

This trial further explored the changes in postoperative cytokine levels in patients with different sleep disorders. The results showed that there was a correlation between severe preoperative sleep disorders and severe postoperative inflammatory reactions. This can be explained by the impact of preoperative sleep disorders on the quality of awakening and pain after general anesthesia. If the patient has sleep disorders before the operation, the sympathetic nerve excitability will increase, and the internal environment homeostasis will be disturbed. At this time, the energy balance regulation mechanism of the body is activated to promote the elevation of proinflammatory factors, reduce the secretion and release inflammatory inhibitory factors, reduce the leukocyte immune activity, and weaken the self-protective effect, thereby aggravating the degree of inflammatory reaction, affecting the adaptability of the body to the stress state. It can delay the recovery time and reduce the quality of recovery, while increasing the incidence of postoperative complications [19]. Therefore, we observed the recovery from anesthesia in patients with different sleep disorders. The results showed that the extubation time and the time when Aldrete score reached 10 in the control group were significantly shorter than that in the other three groups. The time of the two items in group B was significantly longer than that in group A, while that of group C was significantly longer than that in group B. Correlation analysis also showed that extubation time and the time Aldrete score reaching 10 were positively correlated with preoperative PSQI score. All these indicated that the more severe the preoperative sleep disturbance, the worse the postoperative recovery quality. However, in this trial, statistical differences in postextubation complications among the four groups were not found. This result may be related to the use of dexmedetomidine before anesthesia induction and during anesthesia maintenance, as some researchers have found that dexmedetomidine can reduce the incidence of complications after general anesthesia [20].

Sleep disorders can also affect the degree of pain after surgery. Although the mechanism has not been accurately elucidated, one of the known mechanisms is the increase of proinflammatory mediators in patients with sleep disorders, which leads to the aggravation of pain. Zarpelon et al. and Haack et al. have found that IL-6 and TNF-*α* are essential inflammatory mediators of postoperative pain [21, 22].

Significant differences in VAS scores among the four groups at different time points had been discovered in this study. It was proved that the worse preoperative sleep quality and postoperative inflammation were the more severe postoperative pain was, and the time of pain recovery was relatively prolonged. This would have affected the rapid postoperative recovery of patients and worsened the psychological and economic burden of patients.

Nevertheless, some limitations should be acknowledged in this study, such as the small sample size without a priori sample size calculation. In the follow-up study, further expansion of sample size is needed for further observation of the correlation between preoperative sleep disorders and anesthesia recovery.

## 5. Conclusions

In conclusion, the degree of preoperative sleep disorders can affect the postoperative recovery quality and pain of patients undergoing laparoscopic gynecological surgery under general anesthesia, which may be related to the aggravation of inflammatory reaction in the body. Clinicians should pay more attention to the evaluation of the preoperative sleep quality of patients in clinical practice. Utilizing health education, improving the hospitalization environment, and using analgesic and anti-inflammatory drugs or melatonin [23, 24], the patients sleep can be improved, to which will improve the quality of recovery, reduce postoperative pain, and accelerate postoperative rehabilitation.

## Figures and Tables

**Table 1:** Comparison of general data (*n* = 30).

Variables	Control	Group A	Group B	Group C	*F*/*H*	*P*
Age (years)	48.07 ± 10.47	46.20 ± 11.28	53.93 ± 11.19	49.93 ± 9.73	0.749	0.525
BMI (kg/m^2^)	23.15 ± 2.58	23.98 ± 2.65	24.15 ± 2.83	23.62 ± 2.61	0.094	0.963
ASA (I/II)						
I	20	22	19	18	1.297	0.730
II	10	8	11	12		
PSQI (points)	2 (1, 4)	8 (6, 10)	12 (11, 13)	16 (16, 17)	112.380	<0.001

**Table 2:** Comparison of changes in vital signs (*n* = 30).

Variables	Control	Group A	Group B	Group C	*F*/*H*	*P*
MAP (mmHg)						
Preoperative (1 d)	91.73 ± 11.74	92.33 ± 12.07	96.07 ± 10.07	91.81 ± 11.66	0.989	0.401
T1	94.27 ± 7.63	93.40 ± 10.39	97.97 ± 5.78	97.87 ± 10.28	2.239	0.087
T2	81.77 ± 8.69	83.63 ± 11.30	87.18 ± 9.80	86.31 ± 12.82	1.596	0.194
T3	85.83 ± 17.29	90.00 ± 9.65	95.73 ± 10.65	94.80 ± 12.63	1.213	0.308
HR (beat/min)						
Preoperative (1 d)	80.30 ± 11.14	79.87 ± 9.49	80.23 ± 11.09	80.17 ± 9.83	0.010	0.999
T1	75.40 ± 8.70	77.90 ± 9.75	82.53 ± 12.44	80.27 ± 13.34	2.245	0.087
T2	64.07 ± 9.86	67.43 ± 9.96	68.33 ± 12.89	64.40 ± 13.56	1.009	0.391
T3	69.83 ± 9.17	73.77 ± 9.89	75.03 ± 12.94	72.43 ± 11.86	1.213	0.308
Operation time (min)	92.50 (75.00, 115.50)	102.50 (70.00, 155.00)	130.00 (83.75, 141.25)	97.50 (71.25, 131.25)	5.692	0.128
Anesthesia time (min)	105.00 (87.50, 130.00)	122.50 (88.75, 171.25)	145.00 (103.75, 160.00)	115.00 (85.00, 156.25)	5.515	0.138

## Data Availability

The data that support the findings of this study are available on request from the corresponding author.

## Supplementary Materials

Supplementary Materials Supplementary description: Supplementary Figure 1: CONSORT flow chart for patient enrollment.
