# Supplementary figures and images for: Effects of Preoperative Sleep Disorders on Anesthesia Recovery and Postoperative Pain in Patients Undergoing Laparoscopic Gynecological Surgery under General Anesthesia
Source: Mediators Inflamm. 2022 Dec 15;2022:7998104. doi: 10.1155/2022/7998104 (PMC9779992; doi:10.1155/2022/7998104)

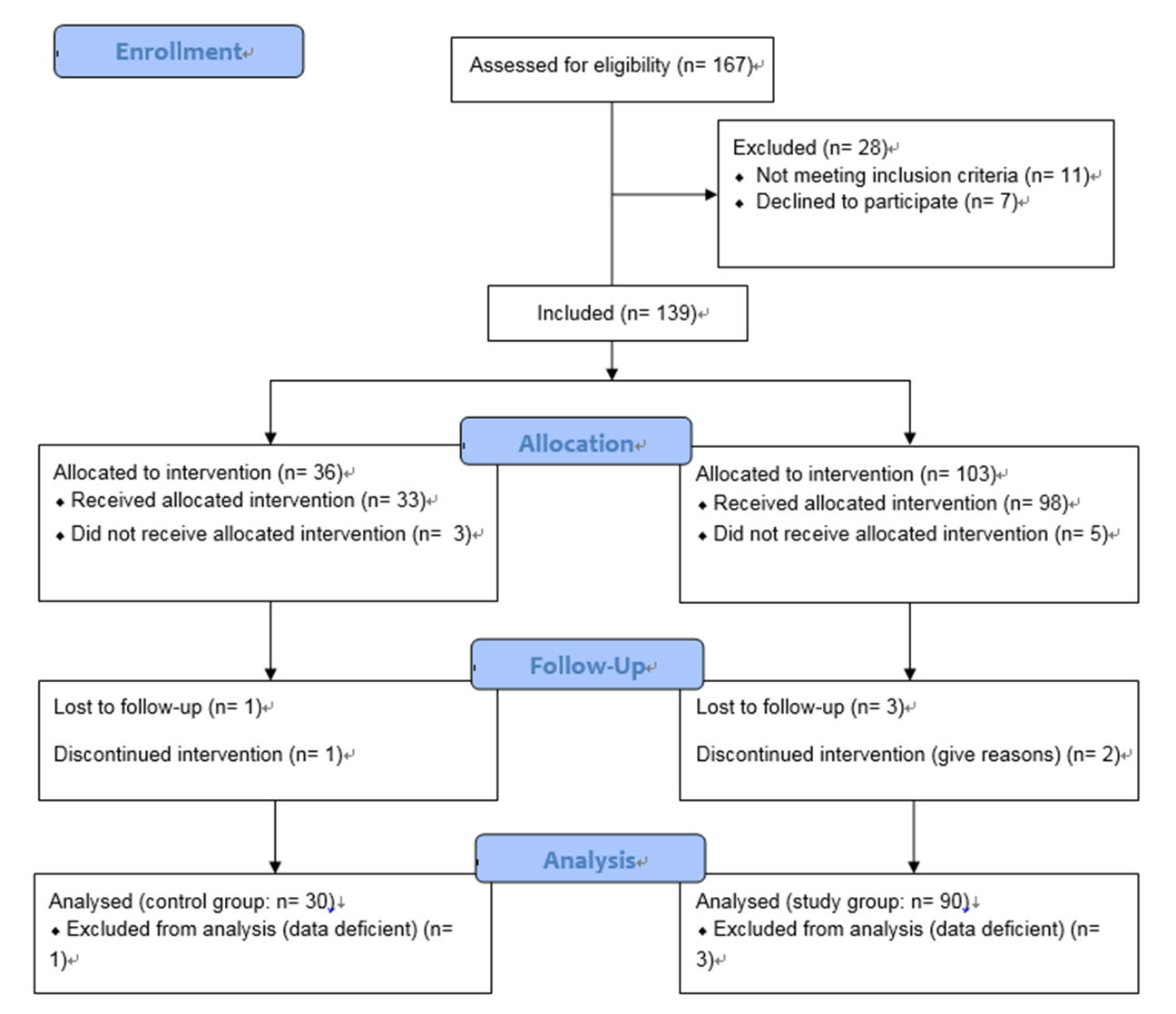


Supplementary Figure 1. CONSORT flow chart for patient enrollment.

Supplement: Supplementary Materials — Supplementary description: Supplementary Figure 1: CONSORT flow chart for patient enrollment. [file 7998104.f1.docx]
